# Assessment of the potential of a reduced dose of dimethyl disulfide plus metham sodium on soilborne pests and cucumber growth

**DOI:** 10.1038/s41598-019-56450-7

**Published:** 2019-12-24

**Authors:** Liangang Mao, Hongyun Jiang, Lan Zhang, Yanning Zhang, Muhammad Umair Sial, Haitao Yu, Aocheng Cao

**Affiliations:** Key Laboratory of Control of Biological Hazard Factors (Plant Origin) for Agriproduct Quality and Safety, Ministry of Agriculture and Rural Affairs of the People’s Republic of China, Institute of Plant Protection, Chinese Academy of Agricultural Sciences, Beijing, 100193 P.R. China

**Keywords:** Small molecules, Small molecules, Soil microbiology, Soil microbiology, Entomology

## Abstract

Methyl bromide (MB), a dominant ozone-depleting substance, is scheduled to be completely phased out for soil fumigation by December 30th 2018, in China. The combined effects of dimethyl disulfide (DMDS) plus metham sodium (MNa) were assessed in controlling soilborne pests for soil fumigation. A study was designed in laboratory for the evaluation of the efficacy of DMDS + MNa to control major soilborne pests. At the same time, two trials were conducted in cucumber field located in Tongzhou (in 2012) and Shunyi (in 2013), respectively, in order to assess the potential of DMDS + MNa in controlling soilborne pests. Laboratory studies disclosed positive synergistic effects of almost all four used combinations on *Meloidogyne* spp., *Fusarium* spp., *Phytophthora* spp., *Abutilon theophrasti* and *Digitaria sanguinalis*. Field trials found that DMDS + MNa (30 + 21 g a. i. m^−2^), both at a 50% reduced dose, effectively suppressed *Meloidogyne* spp. with a low root galling index (2.1% and 11.7%), significantly reduced the levels of *Phytophthora* and *Fusarium* spp. with a low root disease index (7.5% and 15.8%), gave very high cucumber yields (6.75 kg m^−2^ and 10.03 kg m^−2^), and increased income for cucumber growers with the highest economic benefits (20.91 ¥ m^−2^ and 23.58 ¥ m^−2^). The combination treatment provided similar results as MB standard dose treatment (40 g a. i. m^−2^) or DMDS standard dose treatment (60 g a. i. m^−2^) in pest control and yield, but was more effective than MNa standard dose treatment (42 g a. i. m^−2^). Usage of all chemical treatments gave better significant results than the untreated group of control. Considering the economic benefits, the DMDS plus MNa combination (30 + 21 g a. i. m^−2^) could be used for soil fumigation in cucumber production in China.

## Introduction

In northern sites of China, cucumber (*Cucumis sativus* L.) is a leading and important vegetable. Nematodes, soilborne fungi, oomycetes and weeds have a very great potential to lower yield in the protected cucumber production^1,2^. Methyl bromide (MB), an important soil fumigant, has widely been used to control soilborne pathogens, nematodes and weeds in areas of northern China. Though, MB must be completely phased out for soil fumigation by 30 December 2018 in China, because of its deleterious effects on stratospheric ozone^3^. The phasing out of the MB use as a pre-plant soil fumigant has greatly encouraged a deal of research aimed at finding economically acceptable, efficient, and simple alternatives^4^.

Currently, abamectin, fosthiazate, sulfuryl fluoride^2^, metham sodium (MNa) and calcium cyanamide are registered as MB chemical alternatives for cucumber in China. Chloropicrin (Pic)^5^, Dimethyl disulfide (DMDS)^6^, dazomet (DZ)^7^, and 1,3-dichloropropene (1,3-D)^1,8,9^ have been practiced as potential MB alternatives in cucumber. Though, there is no single chemical could replace MB totally^10^. To enhance the control activities for the pests of soilborne and broaden the action spectrum, the mixtures of promising alternative fumigants alone are becoming the scientific research hotspot in recent years. Several soil fumigant combinations, for example, 1,3-D/DZ^7^, 1,3-D/Pic^5,11^, DMDS/DZ^12^, 1,3-D/DMDS^13^ and 1,3-D/MNa^14^ have been tested to control soilborne pests for cucumber production.

Much information could also be retrieved in the literature on combinations of Pic and 1,3-D^11,15–21^, Pic and MNa^21^, 1,3-D and DZ^7,22^, Pic and DMDS^20^, 1,3-D and methyl isothiocyanate (MITC)^23^, Pic and methyl iodide (MeI)^24,25^ and so on as MB chemical alternatives for other crops production. Combinations of DMDS plus DZ have been reported as an efficient alternative to MB in Europe and China^12,22,26^. Both DZ and MNa are MITC generators. The combined use of DMDS and MNa to replace MB, therefore, was at least theoretically promising. Recently, the combined effects of DMDS and MNa were reported in muskmelon^27^ and cut flower^28^. Little information, however, was reported on combination of DMDS plus MNa in cucumber.

Current study was initiated to assess and confirm the efficacy of the combination of DMDS plus MNa on soilborne fungi and oomycetes (*Fusarium* and *Phytophthora* spp.), nematodes (*Meloidogyne* spp.), and weed seeds (*Digitaria sanguinalis* and *Abutilon theophrasti*) under laboratory experiment. Along with, 2 field trials were designed as well, for the determination of the MNa combinations and dimethyl disulfide as an alternative MB for cucumber yield in China at the same time.

## Results

### Laboratory studies

#### Root-knot nematodes

All four applied rates of DMDS + MNa combination tested treatments showed a positive synergistic effect on root-knot nematodes (Table 1), by decreasing the *Meloidogyne* spp. numbers by 90.7%. The maximum practiced dose in our study (80 + 40 mg a.i. kg^−1^) decreased the number of *Meloidogyne* spp. in terms of juveniles by 94.2%.

**Table 1:** Laboratory studies of the combination on root-knot nematodes, soilborne fungi, oomycetes and weed seeds.

Treatment^a^	Rate (g a.i. kg^−1^ soil)	% Corrected nematode mortality				%Control efficacy on *Fusarium* spp.				%Control efficacy on *Phytophthora* spp.				%Control efficacy on *Abutilon theophrasti*				%Control efficacy on *Digitaria sanguinalis*			
		*E*^b^	*E*_0_^c^	*E* - *E*_0_	CE^d^	*E*	*E*_0_	*E* - *E*_0_	CE	*E*	*E*_0_	*E* - *E*_0_	CE	*E*	*E*_0_	*E* - *E*_0_	CE	*E*	*E*_0_	*E* - *E*_0_	CE
DMDS	40	30.1				49.3				20.6				9.5				24.2			
DMDS	80	48.1				57.5				21.9				32.6				33.6			
MNa	20	60.3				56.2				38.8				42.4				11.6			
MNa	40	84.9				59.9				48.2				100.0				56.3			
DMDS + MNa	40 + 20	91.0	72.2	18.8	+	78.5	77.8	0.6	+	62.0	51.4	10.6	+	100.0	47.9	52.1	+	70.8	33.0	37.7	+
DMDS + MNa	40 + 40	92.0	89.4	2.6	+	84.7	79.7	4.9	+	73.9	58.9	15.1	+	100.0	100.0	0.0	±	95.9	66.9	29.0	+
DMDS + MNa	80 + 20	90.7	79.4	11.3	+	90.4	81.4	9.0	+	63.8	52.2	11.6	+	100.0	61.2	38.8	+	100.0	41.3	58.7	+
DMDS + MNa	80 + 40	94.2	92.1	2.1	+	93.6	83.0	10.6	+	74.1	59.5	14.5	+	100.0	100.0	0.0	±	100.0	71.0	29.0	+
**Control**																					

^a^Abbreviations: DMDS = dimethyl disulfide; MNa = metam sodium.

^b^Abbreviations: *E* = the actual control measured efficacy of the combination; data are means of three repeats.

^c^Abbreviations: *E*_0_ = the expected control efficacy of the combination.

^d^Abbreviations: CE = Combined efficacy; If *E* - *E*_0_ > 0, CE was expressed as + ; If *E* - *E*_0_ < 0, CE was expressed as −.

#### Soilborne fungi and oomycetes

Positive synergistic efficacy of four rates of DMDS + MNa tested was also observed on both *Phytophthora* and *Fusarium* spp. (Table 1). The infestation levels through *Fusarium* and *Phytophthora* spp. were decreased by 78.5% and 62.0%, respectively. The maximum applied dose in current study (80 + 40 mg a.i. kg^−1^) decreased *Fusarium* spp. 93.6% and *Phytophthora* spp. 74.1%.

#### Weed control

The four combinations of DMDS + MNa completely controlled the seeds of *Abutilon theophrasti*. However, the combination with the lower rate of MNa only showed synergistic efficacy (i.e. DMDS + MNa at 40 + 20 and 80 + 20 mg kg^−1^); while the combination with the maximum MNa rate did not exhibit synergistic efficacy (i.e. DMDS + MNa at 40 + 40 and 80 + 40 mg kg^−1^) (Table 1). The four practiced rates of DMDS + MNa were all shown synergistic effects on *Digitaria sanguinalis*. The combination with the higher rate of DMDS delivered 100% *Digitaria sanguinalis* control (i.e. DMDS + MNa at 80 + 20 and 80 + 40 mg kg^−1^); the combination with the lower DMDS rate (i.e. DMDS + MNa at 40 + 20 and 40 + 40 mg kg^−1^) provided 70.8% and 95.9% control of *Digitaria sanguinalis*, respectively (Table 1).

### Field trials

#### Root-knot nematodes

The control groups at trial one (in 2012) and trial two (in 2013) were found seriously occupied by *Meloidogyne* spp. (Table 2). The significantly higher levels of *Meloidogyne* spp. were observed in the control group than to all treatments of fumigant (*P* = 0.05).

**Table 2:** Soil fumigation efficacy on numbers of *Meloidogyne* spp. recovered from 100 g of soil, colony-forming units (cfu) *Fusarium* spp. and *Phytophthora* spp. on selective media of 1 g from soil after fumigation, respectively.

Site	Treatment^a^	Rate	*Meloidogyne* spp.		*Fusarium* spp.		*Phytophthora* spp.	
		(g a.i. m^−2^)	No./100 g	% reduction	cfu/g	% reduction	cfu/g	% reduction
Trial one, 2012	DMDS	60	28c^b^	91.5	38c	91.3	953bc	76.5
	MNa	42	114b	65.5	156b	64.2	1820b	55.1
	DMDS + MNa	30 + 21	46c	86.1	28c	93.6	560 cd	86.2
	MB	40	24c	92.7	0d	100	30d	99.3
	Untreated	/	194a	/	436a	/	4053a	/
Trial two, 2013	DMDS	60	38bc	88.5	2570ab	53.4	2178b	70.4
	MNa	42	101b	69.4	1492b	73.0	1778b	75.8
	DMDS + MNa	30 + 21	34bc	89.7	1103b	80.0	1392bc	81.1
	MB	40	13c	96.1	342c	93.8	880c	88.0
	Untreated	/	330a	/	5515a	/	7350a	/

^a^Abbreviations: DMDS = dimethyl disulfide; MNa = metam sodium; MB = methyl bromide.

^b^In each column, data are means of three repeats. Means followed by the same letter are not different (*P = *0.05) according to the LSD test.

The chemical treatments, except for MNa treatment, decreased the nematode infestation levels in trial one and trial two by 86.1% and 88.5%, respectively. The combination treatment of DMDS + MNa suppressed nematode levels by 86.1% and 89.7% in trial one and trial two, respectively; and both results statistically were not found different from MB or DMDS treatment, but higher significantly than MNa treatment at trial one (*P* = 0.05).

#### Soilborne fungi and oomycetes

The groups of control were seriously infested by both *Phytophthora* and *Fusarium* spp. in both trials (Table 2). The levels of infestation with *Fusarium* and *Phytophthora* spp. were observed maximally significant in the control untreated group than to all fumigant treatments (*P* = 0.05) (Table 2).

The lowest *Fusarium* spp. levels were both provided by MB treatment at both trials, followed by the combination DMDS + MNa treatment, which decreased 93.6% *Fusarium* spp. and 80.0% at trial one and two. (Table 2).

Similarly, MB treatment also had the lowest *Phytophthora* spp. level, followed by the DMDS + MNa treatment, and reduced 86.2% and 81.1% *Phytophthora* spp. in trial one and two, respectively (*P* = 0.05) (Table 2). While no statistically change was observed between the MNa + DMDS treatments and MB treatment (*P* = 0.05) (Table 2).

#### The first cucumber fruit yield

The 1st production of cucumber fruit was varied with fumigation treatments (Table S1). Cucumbers that were grown in the untreated control plots provided the minimum yield of first fruiting (0.02 and 0.12 kg m^−2^, respectively) at both field trials (Table S1). No significant change was shown between the chemical treatments at both trials. And the first fruit yield in all chemical treatments were significantly higher than the untreated group of control except for MNa treatment at trial one, 2012 (*P* = 0.05) (Table S1).

#### Cucumber plant height, root galling index and root disease index

The height of cucumber fruit plant at 4 WAT varied with fumigation treatments at both field trials (Table 3). Throughout the chemical treatments at both trials, significantly higher plant height was observed compared to the untreated group of control. And no significant change was shown between the chemical treatments apart from the DMDS treatment at trial one, 2012 (*P* = 0.05) (Table 3).

**Table 3:** Effect of fumigation programs on cucumber plant height, root galling index, and root disease index.

Site	Treatment^a^	Rate (g a.i. m^−2^)	Plant height^b^ (cm)	Root galling Index^c^ (%)	Root disease index (%)
Trial one, 2012	DMDS	60	168.4b^d^	0.7c	20.8b
	MNa	42	182.8ab	28.6b	25.8b
	DMDS + MNa	30 + 21	182.6ab	2.1c	7.5c
	MB	40	196.1a	0.0c	3.3c
	Untreated	/	123.6c	61.4a	50.0a
Trial two, 2013	DMDS	60	180.6a	29.2bc	20.0bc
	MNa	42	181.8a	39.2b	30.8b
	DMDS + MNa	30 + 21	180.6a	11. 7 cd	15.8bc
	MB	40	186.1a	5.8d	4.2c
	Untreated	/	156.3b	78.3a	67.5a

^a^Abbreviations: DMDS = dimethyl disulfide; MNa = metam sodium; MB = methyl bromide.

^b^Cucumber height collected at 4 WAT.

^c^Root galling index and root disease index were collected at the end of the trials.

^d^In each column, data are means of three repeats. Means followed by the same letter are not different (*P = *0.05) according to the LSD test.

By the end of all trials, the nematode infestation was calculated through index of root galling. Root galling index was significantly affected after fumigant treatments (*P* = 0.05). The maximum root galling indexes were both noticed in the untreated plots (61.4% and 78.3% at trials one and two, respectively). At trial one, the lowest index of root galling was provided by MB treatment, which was not different statistically from DMDS + MNa or DMDS alone, but observed lower significantly than MNa alone. At trial two, the minimum root galling index was also gained in MB treated plots, which did not change significantly from DMDS + MNa treatment but was lower significantly than DMDS alone or MNa alone (*P* = 0.05) (Table 3).

While comparing with the untreated control groups, chemical treatments affected root disease index significantly (*P* = 0.05). The maximum index for root disease (50.0% and 67.5%) were provided by the untreated control plots at two trials. At trial one, the lowest root disease index (3.3%) was provided by MB treatment, which was not different statistically from DMDS + MNa (7.5%), but was lower significant than DMDS or MNa alone. At trial two, the lowest root disease index (4.2%) was observed in treated MB plots, which was not significantly different from DMDS + MNa treatment or DMDS alone, but was observed lower than MNa alone, significantly (*P* = 0.05) (Table 3).

#### Cucumber yield, income and economic benefits

The yield of cucumber fruit was differed with chemical treatment (Table 4). Compared with the chemical treatments, yield in control plots was the lowest (3.03 and 5.66 kg m^−2^, respectively, at trials one and two). At trial one, treated plots with DMDS + MNa had the maximum production (6.75 kg m^−2^, 122.8% increase), which was not significantly different from the plots treated with MB (6.66 kg m^−2^, 119.8% increase) or DMDS alone (5.87 kg m^−2^, 93.7% increase), but was significantly higher than MNa alone (4.85 kg m^−2^, 60.1% increase). At trial two, treated MB plots produced the maximum yield (10.12 kg m^−2^, 78.8% increase), that was not found significantly different from the DMDS + MNa treatment (10.03 kg m^−2^, 77.2% increase) or DMDS alone (9.92 kg m^−2^, 75.3% increase), but significantly higher than MNa alone (8.79 kg m^−2^, 55.3% increase) (*P* = 0.05) (Table 4).

**Table 4:** Effect of fumigation programs on cucumber yield, income and economic benefits.

Site	Treatment^a^	Rate (g a.i. m^−2^)	Yield^b^ (Kg m^−2^)	%Yield increase	Gross Income^c^ (¥ m^-2^)	%Income increase	Fumigation cost^d^ (¥ m^-2^)	Net income^e^ (¥ m^-2^)	% Net income increase
Trial one, 2012	DMDS	60	5.87ab^f^	93.7	19.14ab	96.9	1.25	17.89	85.0
	MNa	42	4.85b	60.1	16.93b	74.2	1.60	15.33	58.5
	DMDS + MNa	30 + 21	6.75a	122.8	22.33a	129.9	1.42	20.91	116.2
	MB	40	6.66a	119.8	22.47a	132.0	4.07	18.40	90.3
	Untreated	/	3.03c	/	9.67c	/	0.00	9.67	/
Trial two, 2013	DMDS	60	9.92a	75.3	23.31ab	86.5	1.25	22.06	76.5
	MNa	42	8.79b	55.3	20.05b	60.4	1.60	18.45	47.6
	DMDS + MNa	30 + 21	10.03a	77.2	25.00a	100.0	1.42	23.58	88.6
	MB	40	10.12a	78.8	26.24a	109.9	4.07	22.17	77.4
	Untreated	/	5.66c	/	12.50c	/	0.00	12.50	/

^a^Abbreviations: DMDS = dimethyl disulfide; MNa = metam sodium; MB = methyl bromide.

^b^Collected cucumber yield at each harvest time and summed together at the end of the trials.

^c^Collected cucumber gross income at each harvest time and accumulated together at the end of the trials.

^d^Fumigation cost was the sum of tarp cost and fumigant cost, and the detailed information was listed in Table S2.

^e^Cucumber net income was calculated by the difference of cucumber gross income and fumigation cost.

^f^In each column, data are means of three repeats. Means followed by the same letter are not different (*P = *0.05) according to the LSD test.

Similarly, the gross income of grower through the production of cucumber also varied with chemical treatments (Table 4). Minimum gross income from cucumber cultivation without treatment was obtained (9.67 and 12.50 ¥ m^−2^, respectively). The plots treated with MB provided the highest gross incomes (22.47 and 26.24 ¥ m^−2^, with 132.0% and 109.9% increase, respectively, at trials one and two), which was not different significantly from the DMDS + MNa treatment (22.33 and 25.00 ¥ m^−2^, with 129.9% and 100.0% increase, respectively, at trials one and two) or DMDS alone (19.14 and 23.31 ¥ m^−2^, with 96.9% and 86.5% increase, respectively, at trials one and two), but significantly higher than MNa alone (16.93 and 20.05 ¥ m^−2^, with 74.2% and 60.4% increase, respectively, at trials one and two) (*P* = 0.05) (Table 4).

In addition, the economic benefits (also called net income) of different soil fumigation treatments were assessed. Fumigation cost of different soil treatments were calculated by the sum of fumigants cost and film cost (Table S2). The DMDS + MNa treatment had a relatively low fumigation cost (1.42 ¥ m^−2^), which was lower than MB treatment (4.07 ¥ m^−2^) or MNa alone (1.60 ¥ m^−2^) (Table 4). Net income of growers was finally calculated by the difference of the gross income and fumigation cost (Table 4). In trial one and two, the treated plots with combination DMDS + MNa both had the highest net income (116.2% and 88.6% increase, respectively), followed by MB treated plots (90.3% and 77.4% increase, respectively), DMDS alone treated plots (85.0% and 76.5% increase, respectively), MNa alone treated plots (58.5% and 47.6% increase, respectively) and the untreated plots (*P* = 0.05) (Table 4).

## Discussion

To control root-knot nematode, DMDS is proven equally effective to MB not only in China but elsewhere^16,17,29^, which was confirmed in our both field trials. Anyhow, the overall formulations of DMDS exhibit poor efficacy against soilborne fungi and oomycetes, and moderate activity against weeds. So, additional herbicide and fungicide would be needed to improve soilborne fungi, oomycetes and weed control. MNa, a MITC producer, can control soilborne fungi, oomycetes and weeds efficiently. In the current study, it was observed that the MNa was comparatively less effective ton soilborne fungi and oomycetes than weeds. Thus, the combined use of DMDS and MNa as a promising soil fumigant is at least possible theoretically. All of the combinations of DMDS and MNa in laboratory studies exhibited positive synergy effects on root-knot nematodes, 2 major weed seeds and key soilborne fungi and oomycetes.

The mechanism of specific synergistic activity of DMDS and MNa was still not clearly understood but the laboratory outcomes presented above, preliminarily indicated the possibility of their synergistic usage and affirmed the synergy activity of DMDS and MNa as previously reported by Gerik and Hanson^28^. DMDS is found to inhibit complex IV of mitochondrial electron transport chain (ETC), decrease the intracellular ATP concentration subsequently, which thereby activated neuronal KATP channels mediating membrane hyperpolarization and reduction of neuronal activity^30^. MITC as the active biocidal product of MNa is believed to react with amines and thiols in biological molecules^31^. It will be very difficult to assess a single mechanism of action that accounts for the synergism observed for the combination of DMDS plus MNa. However, two possible mechanisms may offer insight into the synergistic response of the fumigant combination^32^. One mechanism suggested is that sub-lethal exposure of soilborne pests to one fumigant may alter cell processes to such an extent that exposure to a second fumigant is more deleterious than would normally be expected^33^. Another suggested mechanism is that one fumigant causes changes in cell-wall permeability that increases the uptake or decreases the efflux of a second fumigant from the cell^34^. Further understanding of the activity of DMDS and MNa is necessary before the specific synergism mechanism can be deduced.

Our field trials showed that the combined use of DMDS and MNa at 30 + 21 g a.i. m^−2^ rate successfully inhibited *Meloidogyne* spp. root galling, decreased the colony-forming units (cfu) of *Fusarium* spp. sharply and *Phytophthora* spp. on media, sustained maximum production of cucumbers at the same time, and was not different significantly from 40 g m^−2^ MB dose or DMDS alone at 60 g m^−2^ dose, but improved than MNa alone at 42 g m^−2^ dose (*P* = 0.05). The results of field trials above confirmed that the combinations of DMDS and MNa are good choices than alone MB application. Furthermore, we compared the combination of DMDS plus MNa with the used soil fumigant combinations, such as 1,3-D/DZ, 1,3-D/Pic, DMDS/DZ, 1,3-D/DMDS and 1,3-D/MNa (Table S3). Indeed, the combination of DMDS plus MNa is not the best alternative of MB, if only considering the control efficacy. However, considering the application methods and forbidden use issue at the same time, the combination of DMDS plus MNa is more promising than other combinations to replace MB.

Commercial preparations of MNa typically consist of 42% MNa in an aqueous solution at a self-buffered pH > 7. True MNa and halogenated chemicals mixtures (e.g. 1,3-D and Pic) are not available currently due to chemical incompatibility arising when combining them^35,36^. However, the different combinations of DMDS and MNa did not show any degradation according to the synergy effect in our laboratory in the current tests, which may be attributed to the relatively high rates of application^4^.

MNa requires relatively rigorous prerequisites just like DZ for the effective application. The aqueous solution of MNa is diluted by water and applied to the moist soil, where the compound rapidly decomposes to produce MITC. In the vapor and liquid phase of the soil, MITC is toxic to various soilborne pests. However, the physical and chemical properties of MITC (low water solubility, low vapor pressure, and low Henry’s constant) limit its movement and distribution in the soil following MNa application^37,38^. DMDS is highly volatile and a small molecule, so it diffuses and moves in the soil more quickly than MITC^30^. In this point, DMDS could make up the disadvantage of MITC and improve the control efficacy of the combination. Anyhow, uniform distribution in soil is needed by effectual mechanical injection or chemigation system. For better distribution of fumigant in soil, MNa is suitable to be applied by broadcast application or shank injection rather than chisel injection. If there was a good chemigation system available in the field, applying the fumigants by chemigation couldn’t be better.

In concluding remarks, the combination of DMDS and MNa fumigant applied by chemigation was almost equally to MB in terms of controlling knot-nematodes along with soilborne fungus while sustaining high cucumber yields and income for growers. However, much of the detailed work on its application type including its formulations and suitable composites with biological agents (eg. *Trichoderma asperellum*, *Bacillus subtilis* or others) are desirable before the DMDS and MNa combination can be suggested as an efficient substitute to MB for the yield of cucumber in China. The side effects of the combinations on soil beneficial organisms (eg. earthworms, actinomycetes, etc.) should also be considered in further studies.

## Materials and Methods

### Laboratory studies

The combined and single efficacy of DMDS and MNa, was assessed under laboratory study. The collection of soil samples was taken from the 20 cm top in a cucumber field at Shunyi, Beijing, where nematodes and soilborne pests’ presence was abundant. The composition of soil sample was 20.34% sand, 76.25% silt, and 3.41% clay, with pH 6.40 and organic matter content 35.64 g kg^−1^ soil. A soil sieve of 2-mm mesh was used and then mixing was done thoroughly. The moisture of soil was recorded 23.80% (w/w). Analysis for particle size were estimated through pipette method^39^. pH was determined in a 1:2.5 soil to water extract. Organic carbon contents were determined by wet oxidation followed the method of Walkley and Black^40^. Soil moisture contents were performed by remaining the soil at 105 ± 5 °C for 6 hours until constancy of mass was reached^41^.

**Figure 1:**
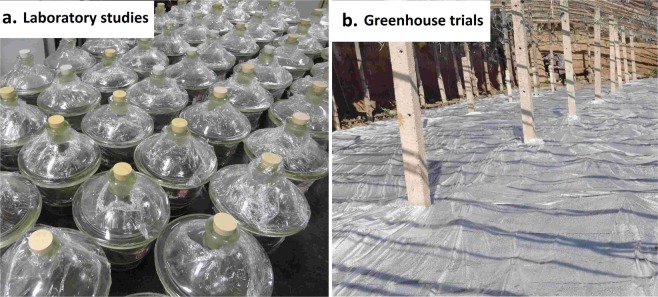
The laboratory study and field trials diagram

Soil samples with six hundred grams weight were placed into each of 27 (2.5 L) desiccators. In each desiccator, 10 *Abutilon theophrasti* seeds and fifteen *Digitaria sanguinalis* seeds were buried at 2 cm depth, respectively. The following fumigant treatments were performed with three replicates: DMDS alone (40, 80 mg a.i. kg^−1^ soil), MNa alone (20, 40 mg a.i. kg^−1^ soil), DMDS + MNa (40 + 20, 40 + 40, 80 + 20, 80 + 40 mg a.i. kg^−1^ soil) and untreated group of control. DMDS or MNa was (Eppendorf, Germany) injected into the soil by pipette, and then the desiccator was sealed immediately. The laboratory study photo (see Fig. 1a.) was taken by the authors. The desiccators were placed at 28°C for five days, and then covers were remained open for one day to release the fumigant residues. The height of weed was then determined. *Fusarium* and *Phytophthora* spp. were both separated from the fumigated soil quantitatively based on the methods of Komada^42^ and Masago *et al*.^43^, respectively. *Meloidogyne* spp. were isolated from one hundred subsample based on Liu methods^44^.

### Field trials

In years 2012 and 2013, two trials demonstration were performed in cucumber growing field at Tongzhou, Beijing and Shunyi, Beijing, respectively, where were both important production areas for cucumber. The farms, which have grown this vegetable for more than five years, are facing serious problems of soilborne pests including soilborne fungi, oomycetes, root-knot nematode, and other pests. Necessary details are given in Tables S4.

99 DMDS TC (Hohhot Guangxin Chemical Trading Co., Ltd., China, containing DMDS of 99%), 42% MNa AS (Shenyang Harvest Agrochemical Co., Ltd., China, a commercial product of 42% MNa) and 98 MB TC (Changyi Chemical Plant, China, containing 98% MB) were selected in the current study. For soil mulch polyethylene film (PE) (0.04 mm thick, from Shouguang Longxing Plastic Co., Ltd., Shandong Province, China) was used.

Randomized blocks design of chemical treatments was performed with three replicates (Table 5). In field trial one, the size of each plot was 17.92 m^2^ (3.2 m wide by 5.6 m long). A treatment as a reference of MB, DMDS alone, MNa alone, DMDS combination with MNa and untreated group of control were established. The application of DMDS was done through chemigation at 60 g a.i. m^−2^ rate; in order to increase the solubility of DMDS in the water, approximate 4% addition of Tween 80 is necessary^13^. MNa was practiced by chemigation at 42 g a.i. m^−2^ rate. Combined treatment of DMDS and MNa was practiced as follows: apply MNa by chemigation at 21 g a.i. m^−2^ rate, and then apply DMDS by chemigation 30 g a.i. m^−2^ rate. To cover the above treatments, PE film was placed. Application of MB between PE sheet and soil was through the hot gas method at 40 g m^−2^ rate. The fumigants were applied on 27 July 2012, and the tarps were removed on 17 August 2012. The cucumbers (cultivar “No.16, Zhongnong”) were transplanted on 28 August 2012, and the final root disease investigation was conducted on 3 December 2012. In field trial two, the size of each plot was 20.16 m^2^ (3.6 m wide by 5.6 m long). All of the treatments in field trial two were the same as those in field trial one (Table 5). The fumigation period was from 10 September 2013 to 26 September 2013. The field trial photo (see Fig. 1b.) was also taken by the authors. The cucumbers (cultivar “No.12, Jinyou”) were transplanted on 5 November 2013, and the final root disease evaluation was conducted on 28 May 2014.

**Table 5:** Experimental Program of the trials.

Sites	Treatment^a^	Rate (g a.i. m^-2^)	Tarp kind^b^	Application methods
Trial one, 2012	DMDS 99 TC	60	PE	Chemigation
	MNa 42 AS	42	PE	Chemigation
	DMDS 99 TC + MNa 42 AS	30 + 21	PE	Chemigation
	MB 98 TC	40	PE	Hot gas method
	Untreated	/	/	/
Trial two, 2013	DMDS 99 TC	60	PE	Chemigation
	MNa 42 AS	42	PE	Chemigation
	DMDS 99 TC + MNa 42 AS	30 + 21	PE	Chemigation
	MB 98 TC	40	PE	Hot gas method
	Untreated	/	/	/

^a^Abbreviations: DMDS = dimethyl disulfide; MNa = metam sodium; MB = methyl bromide; TC = Technical; AS = aqueous solution.

^b^Abbreviations: PE = polyethylene film.

Populations of soil fungi [colony-forming units (cfu) g^−1^ soil] were evaluated after fumigation treatment from soil samples at 0–20 cm depth from the surface of soil. Soil samples were collected from 3 spots in each plot along the diagonal lines. Root-knot nematode densities in the soil were estimated after soil fumigation from 0–20 cm depth. Soil samples from every plot was collected from 3 spots as above. Populations of root-knot nematode and soil fungi were respectively estimated by the same methods as in the laboratory studies.

Height of cucumber plant was determined at 4 weeks after transplant (WAT) (per plot 20 plants). Calculation of root galls and the severity of cucumber root disease were done at the end of the trials (per plot 20 plants). Plants were rated on a scale of 0–4: 0 = 0%, 1 = 1–25%, 2 = 26–50%, 3 = 51–75%, 4 = 76–100% roots galled^4^. Cucumber root disease severity was also rated on a scale of 0−4, 0 = healthy plant and root, without disease; 1 = black brown rotten roots comprise < 25% of the entire root system; 2 = 26−50%; 3 = 51−75%; and 4 = 76−100% black brown rotten roots^45^. Cucumber fruit yields and income of growers were collected after every harvest and summed together at the end of the trials.

### Statistical analyses

#### Laboratory studies

Mortality of nematode was calculated by the equation following.

1 $$X=\frac{{N}_{1}}{{N}_{1}+{N}_{2}}\times 100,$$

where *X* is % nematode mortality, *N*_1_ is the number of dead nematodes, and *N*_2_ is the number of the live nematodes.

Corrected mortality of nematode was considered by the equation following.

2 $$Y=\frac{{X}_{1}-{X}_{2}}{1-{X}_{2}}\times 100,$$

where *Y* is the % corrected nematode mortality, while *X*_1_ is the % nematode mortality of fumigated treatments, and *X*_2_ is the % nematode mortality of untreated control.

Controlling fungi or weed seeds efficacy was measured according to the equation following.

3 $$Y=\frac{{X}_{1}-{X}_{2}}{{X}_{1}}\times 100,$$

where *Y* is the fungi or weed seeds control efficacy, *X*_1_ is the fungal populations or weed height in untreated control group, *X*_2_ is the fungal populations or weed height in treated fumigated plots.

The DMDS plus MNa control efficacy was calculated by the equation following, using methods described by Limpel *et al*.^46^.

4 $${E}_{0}={X}_{1}+{X}_{2}-{X}_{1}{X}_{2}/100,$$

where *E*_0_ is the expected control efficacy of DMDS plus MNa, *X*_1_ is the actual measured control efficacy of DMDS, *X*_2_ is the actual measured control efficacy of MNa. *E* is the actual measured control efficacy of DMDS plus MNa. If *E* > *E*_0_, the combination of DMDS plus MNa was synergistic; if *E* < *E*_0_, the combination of DMDS plus MNa was antagonistic.

#### Field trials

The effectiveness of controlling root-knot nematode or soilborne fungi or oomycetes was measured according to the Eq. (3).

Root galls scores or disease scores recorded for each plot were respectively converted into root galling index (% *RGI*) or root disease indices (% *RDI*) using the formula described by McKinney^45^:

5 $$( \% )RGI/RDI=\frac{\sum (fv)}{NX}$$

where ƒ = number of plants in each class, *v* = class value, *N* = number of observed plants, and *X* = highest value of the evaluation scale.

Statistical software SAS (SAS, version 8.0 for Windows) was run to conduct analysis of variance (ANOVA) for data analysis. Data for populations of soilborne fungus and knot-nematode were transformed as necessary (log10 for large numbers [>100] and square root transformations for small numbers [<100] for statistical analyses), however, all of the data reported here as non-transformed values. Fisher’s LSD test at *P* = 0.05 was used for significant differences determination among means.

## Supplementary information

Supplementary Information
