# Supplementary material for: Assessment of the potential of a reduced dose of dimethyl disulfide plus metham sodium on soilborne pests and cucumber growth
Source: Sci Rep. 2019 Dec 24;9:19806. doi: 10.1038/s41598-019-56450-7 (PMC6930289; doi:10.1038/s41598-019-56450-7)
Supplement: Supplementary file 1 — Supplementary Information [file 41598_2019_56450_MOESM1_ESM.docx]

**Supplementary information**

**Assessment of the potential of a reduced dose of dimethyl disulfide plus metham sodium on soilborne pests and cucumber growth.**

Liangang Mao, Hongyun Jiang, Lan Zhang, Yanning Zhang, Muhammad Umair Sial, Haitao Yu, Aocheng Cao

Key Laboratory of Control of Biological Hazard Factors (Plant Origin) for Agriproduct Quality and Safety, Ministry of Agriculture and Rural Affairs of the People’s Republic of China, Institute of Plant Protection, Chinese Academy of Agricultural Sciences, Beijing, 100193, PR China

Correspondence and requests for materials should be addressed to H.J. (Phone: +8610-62893622; E-mail: [ptnpc@vip.163.com](mailto:ptnpc@vip.163.com))

This supplementary information file contains four supplemental tables.

**Table S1.** Effect of fumigation programs on the first cucumber yield

| Site | Date | Price | Treatment^a^ | Rate | Yield |
| --- | --- | --- | --- | --- | --- |
|  |  | (¥ Kg^-1^) |  | (g a.i. m^-2^) | (Kg m^-2^) |
| Trial one, 2012 | 02/10/12 | 3.2 | DMDS | 60 | 0.21a^b^ |
|  |  |  | MNa | 42 | 0.14ab |
|  |  |  | DMDS + MNa | 30 + 21 | 0.22a |
|  |  |  | MB | 40 | 0.18a |
|  |  |  | Untreated | / | 0.02b |
| Trial two, 2013 | 09/12/13 | 2.0 | DMDS | 60 | 0.29a |
|  |  |  | MNa | 42 | 0.27a |
|  |  |  | DMDS + MNa | 30 + 21 | 0.32a |
|  |  |  | MB | 40 | 0.32a |
|  |  |  | Untreated | / | 0.12b |

^a^ Abbreviations: DMDS = dimethyl disulfide; MNa = metam sodium; MB = methyl bromide.

^b^ In the column, data are means of three repeats. Means followed by the same letter are not different (*P* = 0.05) according to the LSD test.

**Table S2.** Fumigation cost of different soil fumigation treatments in the trials

| Sites | Treatment^a^ | Tarp  kind^b^ | Tarp cost (¥ m^-2^) | Rate  (g a.i. m^-2^) | Preparation rate (Kg m^-2^) | Fumigant Price (¥ Kg^-1^) | Fumigant cost (¥ m^-2^) | Total cost^c^ (¥ m^-2^) |
| --- | --- | --- | --- | --- | --- | --- | --- | --- |
| Trial one, 2012 | DMDS 99 TC | PE | 0.40 | 60 | 0.061 | 14 | 0.85 | 1.25 |
|  | MNa 42 AS | PE | 0.40 | 42 | 0.100 | 12 | 1.20 | 1.60 |
|  | DMDS 99 TC + MNa 42 AS | PE | 0.40 | 30 + 21 | 0.030+0.050 | 14+12 | 1.02 | 1.42 |
|  | MB 98 TC | PE | 0.40 | 40 | 0.041 | 90 | 3.67 | 4.07 |
|  | Untreated | / | 0.00 | / | / | / | 0.00 | 0.00 |
| Trial two, 2013 | DMDS 99 TC | PE | 0.40 | 60 | 0.061 | 14 | 0.85 | 1.25 |
|  | MNa 42 AS | PE | 0.40 | 42 | 0.100 | 12 | 1.20 | 1.60 |
|  | DMDS 99 TC + MNa 42 AS | PE | 0.40 | 30 + 21 | 0.030+0.050 | 14+12 | 1.02 | 1.42 |
|  | MB 98 TC | PE | 0.40 | 40 | 0.041 | 90 | 3.67 | 4.07 |
|  | Untreated | / | 0.00 | / | / | / | 0.00 | 0.00 |

^a^ Abbreviations: DMDS = dimethyl disulfide; MNa = metam sodium; MB = methyl bromide; TC = Technical; AS = aqueous solution.

^b^ Abbreviations: PE = polyethylene film.

^c^ Total fumigation cost was the sum of tarp cost and fumigant cost.


**Table S3**. Comparation of the used soil fumigant combinations with DMDS plus MNa.

| Soil fumigant combinations^a^ | Application methods | Forbidden use issue | Control efficacy on various soilborne pest in cucumber |
| --- | --- | --- | --- |
| DMDS/MNa | Easy (Chemigation, successively) | None | 81.1%~93.6% |
| DMDS/DZ | **Complex (Injection + soil mixture, respectively)** | None | 84.5%~97.6% |
| 1,3-D/DMDS | Easy (Injection or chemigation) | **1,3-D is banned in the Europe** | 72.4%~99.2% |
| 1,3-D/Pic | Easy (Injection) | **1,3-D is banned in the Europe** | 86.3%~97.5% |
| 1,3-D/DZ | **Complex (Injection or chemigation + soil mixture, respectively)** | **1,3-D is banned in the Europe** | 84.5%~100% |
| 1,3-D/MNa | **Complex (Chemigation + Chemigation, with** **an interval of approximately one week)** | **1,3-D is banned in the Europe** | 89.9%~100% |

^a^ Abbreviations: DMDS = dimethyl disulfide; MNa = metam sodium; DZ = dazomet; 1,3-D = 1,3-dichloropropene; Pic = chloropicrin.

**Table S4.** Soil characteristics of the experimental sites

| Site | Organic matter  (g kg^-1^) | Available K  (mg kg^-1^) | N/NH_4_^+^  (mg kg^-1^) | Available P  (mg kg^-1^) | N/NO_3_^-^  (mg kg^-1^) | pH  (1:2.5) | Bulk density  (g·cm^-3^) | Soil moisture  (%) |
| --- | --- | --- | --- | --- | --- | --- | --- | --- |
| Trial one, 2012 | 30.1 | 574.6 | 33.5 | 383.0 | 336.9 | 7.87 | 0.90 | 30.1 |
| Trial two, 2013 | 32.0 | 1069.0 | 3.2 | 578.0 | 53.6 | 6.87 | 0.91 | 22.0 |
